# Impact of Gut Microbiota on Bone Metabolism—Present Concepts and Therapeutic Implications

**DOI:** 10.3390/ijms27093865

**Published:** 2026-04-27

**Authors:** Marta Toboła, Alina Kuryłowicz

**Affiliations:** Department of Internal Medicine and Geriatric Cardiology, Centre of Postgraduate Medical Education, Orlowski Hospital, 00-416 Warsaw, Poland; marta.tobola1@gmail.com

**Keywords:** microbiome, calcium homeostasis, bone metabolism, dysbiosis, osteoporosis

## Abstract

The gut microbiota plays a multifaceted role in calcium homeostasis and bone metabolism —acting through metabolic, immunological, and hormonal pathways that collectively constitute the gut–bone axis. The microbiota influences calcium bioavailability through several overlapping mechanisms that act in the intestine. Moreover, microbial fermentation products may directly impact the osteoblast–osteoclast interplay and, by modulating immune and endocrine functions, are crucial for bone metabolism. A healthy microbiota supports bone formation; however, intestinal dysbiosis may impair bone structure and function. This narrative review aims to present pathways linking the gut microbiota to bone metabolism, both in health and disease. First, we will discuss the influence of gut microbiota on calcium absorption. We will then outline the role that microbial metabolites, such as bile acids and short-chain fatty acids (SCFAs), play in regulating bone structure and function. In the following section, we will discuss the role of the microbiota in the immunological and hormonal modulation of bone metabolism. Finally, we will discuss how dysbiosis affects bone and how therapeutic interventions, such as probiotics, prebiotics, and postbiotics, may influence bone tissue quality.

## 1. Introduction

Recent years have shown that the human microbiota, composed of trillions of symbiotic microorganisms residing primarily in the gastrointestinal tract, on the skin, and at mucosal surfaces, plays a critical role in maintaining host homeostasis [1]. Among these, the gut microbiota plays a significant role by stimulating nutrient synthesis, producing short-chain fatty acids (SCFAs), and regulating immune function, whilst also acting as an essential protective barrier against opportunistic pathogens [2]. Consequently, microbial dysbiosis disrupts these physiological functions and significantly impacts the pathogenesis of many localised and systemic diseases, including metabolic, inflammatory and neurobehavioural disorders [3].

The skeleton is a constantly remodelling organ that maintains a dynamic equilibrium between bone formation and resorption [4]. These processes are governed by a dynamic interplay of local cellular signals, systemic endocrine hormones, and mechanical stimuli to maintain skeletal structural integrity and fulfil systemic mineral and metabolic demands [5]. Both descriptive and interventional data show that the gut microbiota also plays a multifaceted role in bone metabolism: acting through metabolic, immunological and hormonal pathways that collectively constitute the gut–bone axis [6]. Furthermore, recent Mendelian randomisation studies have provided evidence of causal relationships, rather than associations, between gut microbial taxa and calcium–phosphorus homeostasis and metabolic bone diseases [7].

Metabolic bone diseases, predominantly osteoporosis, represent a significant epidemiological challenge that imposes substantial morbidity, mortality and socioeconomic burdens on geriatric populations worldwide [8]. These osteometabolic conditions are characterised by the progressive deterioration of bone microarchitecture and reduced bone mineral density (BMD), culminating in low-trauma fragility fractures [9]. Given the gut microbiota’s ability to modulate bone metabolism, modifying the microbiome might represent a therapeutic strategy for treating metabolic bone diseases [10].

This narrative review aims to present concepts regarding pathways linking the gut microbiota to bone metabolism, both in health and disease. Therefore, we discuss conceptions regarding the influence of gut microbiota on calcium absorption. We then present data on the role of microbial metabolites, such as bile acids and SCFAs, in regulating bone structure and function. We will then discuss the role of gut microbiota in the immunological and hormonal modulation of bone metabolism. Finally, we will outline how dysbiosis affects bone and how therapeutic interventions, such as probiotics, prebiotics and postbiotics, may influence bone tissue quality.

## 2. Methods

To summarize findings regarding the effects of gut microbiota on bone metabolism, a literature search was performed in the PubMed database. Articles used for this review addressed the impact of the gut microbiota and its metabolites on calcium absorption and bone metabolism across three experimental setups (cell, animal, and human studies), as well as data on the potential therapeutic use of pro-, pre-, and postbiotics in metabolic bone diseases. A systematic literature search was conducted from 2001 to 2026 using the following terms: “gut microbiota” OR “bile acids” OR “short-chain fatty acids” OR “dysbiosis” OR “probiotics” OR “prebiotics” OR “postbiotics” combined with “calcium absorption” OR “bone metabolism” OR “osteoporosis”. Only articles published in peer-reviewed scientific journals were included in the analysis. Of the 471 identified papers, after excluding titles and abstracts that did not meet the inclusion criteria, 90 unique articles were included in this mini review (Supplementary Table S1).

## 3. Impact of Gut Microbiota on Calcium Absorption

**Figure 1:**
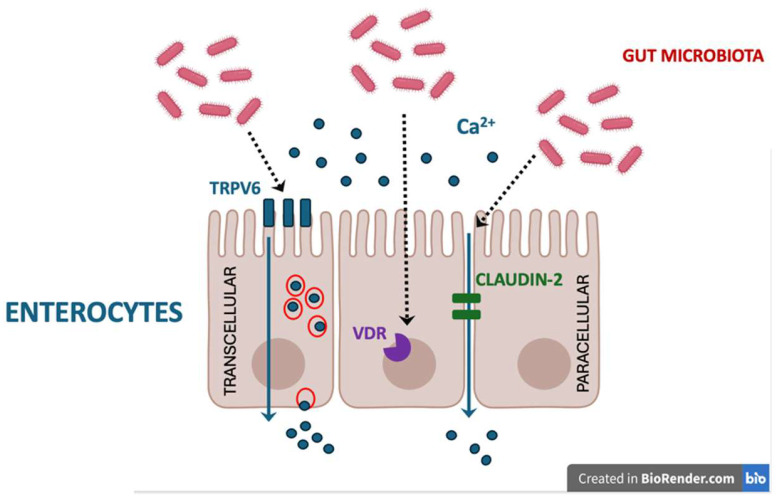
The gut microbiota and its metabolites may influence calcium bioavailability through several overlapping mechanisms, including (among others): (i) increasing the pool of bioavailable ionised calcium (Ca^2+^) in the gut; (ii) upregulating the expression of TRPV6 while simultaneously facilitating passive paracellular diffusion by modulating tight junction proteins (e.g., claudin-2); (iii) increasing the expression of the intestinal vitamin D receptor (VDR), enhancing the effects of vitamin D. Created in BioRender. Kurylowicz, A. (2026) https://BioRender.com/1jiu4kr.

The pleiotropic impact of the gut microbiota on bone metabolism involves influencing calcium bioavailability through several overlapping mechanisms [Table 1, Figure 1]. Firstly, the fermentation of prebiotics by bacteria such as *Bifidobacterium* and *Lactobacillus* yields SCFAs (e.g., acetate, propionate, and butyrate) that may enhance calcium solubility and promote its transport across the colonic epithelium [11]. These organic acids lower the local pH in the cecum and colon, which prevents the formation of insoluble calcium–phosphorus complexes. The increased acidity promotes the ionisation of minerals into ${\mathrm{Ca}}^{2+}$, thereby expanding the pool of bioavailable calcium for distal gut absorption, as shown in in vitro models [11,12,13]. The same mechanism is likely involved in the increased calcium absorption induced by SCFAs in humans [14].

Moreover, SCFAs serve as the primary energy substrate for healthy colonocytes and stimulate crypt cell proliferation and enterocyte hypertrophy, thereby significantly expanding the total mucosal surface area available for calcium absorption in the intestine, as shown in both animal models and humans [15,16]. Subsequently, the beneficial effect of SCFAs on bone remodelling has been linked to increased calcium (and magnesium) absorption after SCFAs supplementation in clinical trials [17].

Gut microbiota influence the active routes of calcium transport in the intestine, too. The active transcellular pathway involves apical calcium entry via the transient receptor potential vanilloid 6 (TRPV6) channel and intracellular transport by Calbindin-D9k. In animal models, butyrate supplementation directly upregulates the expression of TRPV6 and Calbindin-D9k, while simultaneously facilitating passive paracellular diffusion by modulating tight junction proteins (e.g., claudin-2) [18].

The intestinal microbiota may also indirectly affect calcium absorption by regulating bile acid metabolism: certain bacterial strains that possess 7β-hydroxysteroid dehydrogenase (e.g., *Ruminococcus gnavus*) convert primary bile acids into secondary bile acids, which promote calcium absorption by increasing intestinal vitamin D receptor expression [19,20]. Furthermore, a balanced microbial flora maintains intestinal integrity, supporting optimal vitamin D absorption, a prerequisite for transcellular calcium uptake, as calcitriol has been shown to upregulate *TRPV6* expression in a human colon adenocarcinoma cell line (Caco-2 Cells) [21].

As a result, a microbiota-driven increase in calcium absorption suppresses parathormone (PTH) secretion, thereby blunting osteoclast activity and preventing bone matrix resorption. In states of metabolic or hormonal dysregulation, prebiotic interventions that stimulate SCFA production effectively mitigate bone demineralisation and improve structural density.

**Table 1:** Mechanisms by which the gut microbiota influences calcium absorption in the intestines.

Calcium Transport Pathway [Ref]	Anatomic Location	Microbial Influence	Key Mediators Involved
active transcellular [18,19,20,21]	duodenum and jejunum	SCFAs upregulate transcription of apical channels and binding proteins secondary bile acids	TRPV6, Calbindin-D9k, VDR
passive paracellular [11,12,13]	entire intestine	acidification increases luminal ionic calcium concentration, driving passive diffusion	soluble ${\mathrm{Ca}}^{2+}$, intercellular tight junctions

SCFAs—short-chain fatty acids; TRPV6—transient receptor potential vanilloid 6 channel; VDR—vitamin D receptor.

## 4. The Role of Microbial Metabolites: Bile Acids and SCFAs in Regulating Bone Metabolism

### 4.1. Bile Acids

One mechanism by which the gut microbiota regulates bone metabolism is the conversion of primary bile acids into secondary bile acids, which then act as systemic signalling molecules in the endocrine system.

Once in the gastrointestinal tract, the gut microbiota utilises specific enzymes to deconjugate and dehydroxylate primary bile acids, converting them into secondary bile acids such as lithocholic acid and hyodeoxycholic acid (HDCA) [22]. This biotransformation process alters the systemic bile acid pool, thereby influencing distant physiological systems, including the skeleton [23]. The gut bacteria shape the bile acid pool through diverse enzymes, beginning with bile salt hydrolase/transferase (BSH/T), which bidirectionally regulates conjugation and deconjugation. Following this initial gateway, the highly conserved *bai* operon drives a multi-enzyme 7α-dehydroxylation cascade that converts primary bile acids into highly hydrophobic secondary variants. Further structural diversity is generated by hydroxysteroid dehydrogenases (HSDHs) and 5α/β-reductases, which catalyse epimerization and reduction reactions to produce epimeric and iso-variants with distinct immunomodulatory and antimicrobial properties. Additionally, specialised microbial enzymes, such as sulfotransferases and acyl synthetases, produce sulfated and acylated bile acid derivatives [24]. However, many of these complex modifications remain unmapped, and our understanding of the gut microbiota’s role in bile acid modification remains incomplete.

Preclinical studies suggest that circulating secondary bile acids directly influence bone homeostasis by activating two main receptors: the nuclear farnesoid X receptor (FXR) and the membrane G protein-coupled receptor TGR5 (Table 2). Both receptors are expressed in bone tissue, and their activation status is highly dependent on the composition of the gut microbiota and its resulting bile acid profile. Activation of FXR by bile acids stimulates osteoblast differentiation via the Wnt/β-catenin and extracellular signal-regulated kinase (ERK) signalling pathways in mouse ST-2 mesenchymal stem cells, directly promoting bone mineralisation in animal models [25,26]. In addition, in other experimental settings (cervical cancer cells), FXR signalling inhibits nuclear factor κB (NF-κB)—a chief trigger of proinflammatory responses involved in bone resorption [27,28]. Simultaneously, the interaction between secondary bile acids such as HDCA and the TGR5 receptor strongly inhibits osteoclastogenesis by blocking the nuclear translocation of P65 and modulating AMP-activated protein kinase (AMPK) signalling, thereby preventing excessive bone resorption in mice model of osteoporosis [29]. Dysbiosis disrupts the normal bile acid pool, impairing receptor signalling and contributing to pathological bone loss [24].

**Table 2:** Bile acid receptors key for bone metabolism.

Receptor	Primary Cellular Target	Molecular Mechanism [Ref]	Overall Skeletal Effect [Ref]
FXR	Osteoblasts Osteoclasts	Promotes Wnt/β-catenin and ERK signaling [25]; inhibits NF-κB [27]	Enhances bone formation and suppresses resorption [26]
TGR5	Osteoclasts	Activates AMPK signaling; blocks P65 nuclear translocation [29]	Strongly inhibits osteoclast maturation and activity [29]

AMPK—AMP-activated protein kinase, ERK—extracellular signal-regulated kinase, FXR—farnesoid X receptor, NF-κB—nuclear factor κB, TGR5—membrane G protein-coupled receptor.

### 4.2. SCFAS

In addition to facilitating calcium absorption, SCFAs can be absorbed in the intestine and enter the systemic circulation, thereby exerting a direct influence on bone metabolism. Interestingly, preclinical studies suggest that the effects of SCFAs on bone are not unambiguous and depend on the mechanism by which they act.

In this context, it is worth noting that they primarily act through specialised G protein-coupled receptors (GPCRs) that detect metabolites produced by the gut microbiota’s fermentation of dietary fibre. Table 3 presents examples of GCPRs that have been shown to mediate the effects of SCFAs on bone metabolism. Overall, the research suggests that, by acting via GPCRs, SCFAs tend to stimulate osteoclastogenesis and inhibit the differentiation of mesenchymal cells into osteoblasts [30,31,32,33].

However, SCFAs also act as histone deacetylase (HDAC) inhibitors: by inhibiting class I and class II HDACs, they promote histone hyperacetylation, which alters gene expression and, in turn, induces cell cycle arrest and differentiation [34]. In the MC3T3-E1 preosteoblast line, butyric acid, by causing excessive acetylation of histone H3, induced a temporary increase in osteoblast proliferation and viability, but did not affect the cell cycle [35]. In turn, human amniotic membrane-derived mesenchymal stem cells (hAMSCs) were inhibited in proliferation by low concentrations of butyrate, which arrested the cell cycle in the G0/G1 phase. Next, during osteogenic induction, butyrate at a concentration of <1.0 mM for 3 days induced expression of osteogenic genes. However, a higher concentration (1.0 mM) and longer exposure time (14 days) to butyrate did not produce such effects, which can partly be attributed to increased expression of histone deacetylase 8 (HDAC8), thereby inhibiting transcription during osteogenesis [36]. Moreover, higher concentrations of SCFAs and longer exposure times can induce cytotoxicity, enhance receptor activator of NF-κB ligand (RANKL) and reduce osteoprotegerin expression in osteoblast cultures [37,38].

**Table 3:** Key short-chain fatty acids (SCFAs) G protein-coupled receptors (GPCRs), and their potential role in bone metabolism.

Type of Receptor	Description	Potential Role in Bone Metabolism	References
FFA2 (GPR43)	Activated by acetate, propionate, and butyrate; plays a key role in immune response regulation and intestinal health	• GPR43-mediated signaling: ‑ Promotes adipogenic differentiation of mesenchymal stem cells in vitro ‑ Stimulates osteoblastogenesis and bone formation in animal models	[31,32]
FFA3 (GPR41)	Primarily activated by propionate and butyrate; involved in mediating effects of SCFAs on enteroendocrine cells and gut–brain communication	• GPR41-mediated signaling: ‑ Regulates bone mass by directing the differentiation of mesenchymal stem cells towards adipocytes rather than osteoblasts ‑ Reduces mineralisation and promotes bone marrow adiposity • Double Gpr41/43 double knockout mice exhibit increased trabecular bone mass	[32]
GPR109a (HCAR2)	Recognized as a receptor for butyrate and niacin; acts as a tumor suppressor in the colon and regulates inflammatory responses	• Deletion of *GPR109A* gene: ‑ Exerts inhibitory effect on osteoclastogenesis in vitro ‑ Leads to increased bone mass in animal models • GPR109A -mediated signaling: ‑ Increases insulin-like growth factor 1 secretion in the liver and therefore indirectly impacts bone formation	[33,39,40]

Another mechanism by which SCFAs may impact bone function involves their stimulatory effect on insulin-like growth factor 1 (IGF-1) secretion. Specifically, butyrate activates the GPR109A receptor in hepatic Kupffer cells, thereby increasing interleukin-6 (IL-6) secretion, which subsequently stimulates IGF-1 production in the liver. Furthermore, HDAC inhibition regulates the secretion of IGF-binding proteins (IGFBPs) in intestinal epithelial cells, thereby modulating the bioavailability of circulating free IGF-1 [39]. IGF-I is required for maintaining the normal interaction between the osteoblast and osteoclast to support osteoclastogenesis through its regulation of RANKL and RANK expression [40]. In experimental models, antibiotic-induced depletion of gut microbiota reduces both SCFA levels and serum IGF-1, while targeted SCFA supplementation fully restores IGF-1 concentrations and promotes bone formation [41].

## 5. Role of Gut Microbiota in Immune Modulation of Bone Metabolism

The gut microbiota composition and its derived metabolites influence systemic inflammation and immune cell differentiation, thereby shaping systemic immune tone and directly affecting bone remodelling. The best examples of this phenomenon are germ-free mouse studies, which demonstrate drastically reduced osteoclast populations and lower levels of pro-inflammatory mediators (IL-6, tumour necrosis factor α (TNF-α), RANKL), which are restored upon microbiota reconstitution [42]. Table 4 summarises the roles of Treg and Th17 cells in regulating bone metabolism.

**Table 4:** Role of Th17 and T regulatory (Treg) cells in the regulation of bone metabolism.

T Cell Subtype	Primary Cytokines Secreted	Impact on Bone Metabolism [References]
Th17 Cells	IL-17, TNF-α	Promotes RANKL expression and drives osteoclastogenesis [43]
Treg Cells	IL-10, TGF-β	Suppresses inflammation and counteracts bone resorption [44]

IL—interleukin, RANKL—receptor activator for nuclear factor κB ligand, TGF-β1—transforming growth factor β1, TNF-α—tumor necrosis factor alpha.

The best-characterised mechanism by which the microbiota regulates bone remodelling via the immune system is the modulation of T-cell populations, specifically the balance between regulatory T (Treg) and T helper 17 (Th17) cells [43,44].

The key regulators of the balance among T lymphocyte subpopulations are SCFAs and other microbiota-derived bioactive metabolites, such as bile acids and tryptophan derivatives, which cross the intestinal barrier to exert systemic immunomodulatory effects. Butyrate-mediated induction of Treg cells can prevent osteoclast formation by secreting osteoclastogenic cytokines, such as interleukin (IL) 10, and through cell–cell contact-dependent interactions (e.g., CTLA4-CD80/CD86) [45]. However, this mechanism does not appear to be crucial for maintaining proper osteoblast and osteoclast differentiation, as Rag1-knockout mice (lacking T and B cells) still exhibit increased bone density following treatment with propionate and butyrate [45]. Tryptophan metabolites (e.g., indole-3-acetic acid (IAA) and its derivatives) can promote the differentiation and function of Tregs as well [46]. The same applies to bile acid metabolites, which regulate the balance of mitochondrial reactive oxygen species and promote Treg generation [47].

T helper 17 (Th17) cells are a diverse population classified into subtypes with distinct non-pathogenic and pathogenic characteristics depending on the surrounding cytokine environment. In the presence of IL6 and transforming growth factor β1 (TGF-β1), naive CD4^+^ T cells differentiate into non-pathogenic Th17 cells. In contrast, exposure to IL-6, IL-23, and IL-1β generates pathogenic Th17 cells that produce IL-17, interferon-γ (IFN-γ), and granulocyte-macrophage colony-stimulating factor (GM-CSF), and induce several autoimmune diseases [48]. Microbiome metabolites modulate the differentiation and activity of Th17 cells, as demonstrated in numerous in vitro and in vivo models. SCFAs such as butyrate, by inhibiting HDACs, epigenetically downregulate the expression of the master transcription factor retinoic acid-related orphan receptor gamma t (RORγt), which is essential for Th17 lineage commitment, and subsequently reduce the production of pro-inflammatory cytokines like interleukin-17 (IL-17) [49,50,51]. Inhibition of RORγt is also a mechanism by which bile acid metabolites, such as isoallolithocholic acid, suppress Th17 cell differentiation [47,52]. In addition to epigenetic modifications, SCFAs directly alter the intracellular metabolic pathways of Th17 cells. By inducing acetylation of p70 S6 kinase, SCFAs modulate the mechanistic target of rapamycin (mTOR) pathway, which is an essential step in T-cell differentiation [50]. Furthermore, specific SCFAs, such as pentanoate, increase glycolytic activity and the rate of extracellular acidification in Th17 cells [53]. These metabolic changes, in conjunction with HDAC inhibition, drive the transdifferentiation of pathogenic Th17 cells into a non-pathogenic, regulatory IL-10-producing phenotype. The impact of SCFAs on Th17 cells is plastic and depends on the prevailing cytokine milieu. Under autoimmune conditions, SCFAs predominantly inhibit Th17 polarization to mitigate tissue inflammation. However, during active infections with robust pro-inflammatory signalling, SCFAs can paradoxically support the generation of effector Th17 cells to combat pathogens. Notably, even as they promote effector expansion, SCFAs concurrently induce IL-10 expression to prevent excessive immune-mediated tissue damage [54]. In turn, the impact of tryptophan metabolites on Th17 proliferation and function is less unambiguous. On the one hand, IAA can decrease Th17 cell activation by interacting with the aryl hydrocarbon receptor (AhR); on the other hand, 6-formylindolo(3,2-b)carbazole has been shown to promote T cell differentiation into Th17 cells [55].

The data presented above are derived primarily from experimental settings. Observational studies show that commensal microbes can sway the Tregs/Th17 equilibrium; for instance, *Bifidobacterium* species promote regulatory Treg differentiation in the bone marrow and spleen while suppressing excessive bone resorption [56]. Conversely, pathogenic bacteria, including segmented filamentous bacteria and certain *Ruminococcus* species, exacerbate osteoclastogenesis and bone catabolism by activating Th17 cells [57].

## 6. Impact of Microbiota on Hormonal Regulation of Bone Metabolism

Another mechanism by which the gut microbiota can influence bone metabolism is its modulatory effect on the endocrine system. In addition to the interaction with IGF-1 described above, gut microbiota has a significant effect on the synthesis and action of parathyroid hormone (PTH), as well as on vitamin D and vitamin K2.

### 6.1. Parathyroid Hormone

The gut microbiota influences parathyroid hormone (PTH) secretion primarily by modulating intestinal calcium absorption (as described above). The literature also includes data suggesting that microbial metabolites may act as positive allosteric modulators of the calcium-sensing receptor (CaSR) on parathyroid cells. These CaSR modulators include aromatic and aliphatic amino acids, particularly L-phenylalanine and L-tryptophan, as well as polyamines (e.g., spermine, spermidine). Upon entering the systemic circulation, these endogenous polycationic amines and amino acids may bind to specific allosteric clefts near the orthosteric site within the amino-terminal domain of the parathyroid CaSR, acting as natural calcimimetics [58]. This subsequently induces a conformational shift that enhances the receptor’s sensitivity to physiological levels of extracellular ionised calcium and suppresses the vesicular exocytosis of PTH from chief cells [59]. However, further research is needed to understand the role of microbial metabolites in CaSR sensitisation.

In addition, gut microbiota metabolites determine the proper action of PTH in bone tissue. Microbially derived butyrate binds to GPR43 receptors on immune cells, stimulating expression of Wnt10b (Wingless-type MMTV integration site family, member 10B)—a gene that encodes a member of the Wnt family of secreted, lipid-modified signalling glycoproteins, necessary for PTH-induced bone anabolism. Conversely, in hyperparathyroidism, segmented filamentous bacteria drive the expansion of intestinal Th17 cells that migrate to the bone marrow, thereby enabling PTH-mediated bone resorption [60].

### 6.2. Vitamin D

The relationship between gut microbiota and vitamin D is bidirectional. On the one hand, the gut microbiota may facilitate the absorption and enzymatic activation of vitamin D. On the other hand, vitamin D and its receptor critically shape microbial composition, immune homeostasis, and intestinal barrier integrity [61].

The formation of the active metabolite of vitamin D, calcitriol, depends on the hepatic and renal hydroxylation of cholecalciferol. The gut microbiota may play a role in these processes. Certain bacterial strains possess their own cytochrome P450-like enzymes, such as CYP105A1 found in *Streptomyces griseolus*, which can directly hydroxylate cholecalciferol into its biologically active forms [62]. However, the significance of this phenomenon in the formation of the systemic pool of active vitamin D metabolites in humans remains unknown.

The microbiome may also regulate host endocrine vitamin D metabolism by modulating the systemic expression of fibroblast growth factor 23 (FGF23), a hormone that controls the renal synthesis of active 1,25-dihydroxyvitamin D [63]. High microbial diversity, particularly the presence of butyrate-producing bacteria, is strongly correlated with increased systemic levels of active vitamin D [64]. Furthermore, gut bacteria synthesise secondary bile acids and mediate lipid metabolic pathways essential for incorporating fat-soluble vitamin D into mixed micelles, which is a prerequisite for its intestinal absorption [61].

Adequate vitamin D signalling via its receptor (VDR) increases microbial α-diversity, promotes beneficial butyrate-producing bacteria, and helps optimise the *Firmicutes-to-Bacteroidetes* ratio. In addition, VDR activation upregulates the expression of epithelial tight junction proteins and mucins in goblet cells, thereby preventing increased intestinal permeability and bacterial translocation [61]. Impaired vitamin D signalling degrades gut barrier function, leading to the leakage of gut-derived endotoxins, such as lipopolysaccharides, into the bloodstream [65].

### 6.3. Vitamin K

Various bacterial genera residing in the human gastrointestinal tract, including *Bacteroides*, *Escherichia*, *Lactobacillus*, and *Bifidobacterium*, can synthesise vitamin K2 (menaquinone). These microbes convert dietary precursors and ferment indigestible fibres to produce various menaquinone subtypes, including MK-7, MK-8, and MK-10. The bacterially produced menaquinones are then absorbed through the intestinal lining, particularly in the ileum and colon, providing a vital endogenous source that supplements dietary vitamin K intake [66]. Vitamin K2 serves as a critical cofactor for the enzyme γglutamyl carboxylase, which facilitates the γcarboxylation of osteocalcin. Osteocalcin is a protein secreted by osteoblasts that, in its active carboxylated form (cOC), binds strongly to calcium ions and hydroxyapatite within the extracellular matrix. This binding process directly drives bone mineralisation and strengthens the skeletal structure, whereas high levels of inactive, uncarboxylated osteocalcin (ucOC) indicate vitamin K deficiency and elevated fracture risk [67,68].

Data from preclinical studies suggest that beyond protein carboxylation, microbiota-derived vitamin K2 actively modulates the cellular dynamics of bone remodelling. It promotes osteoblastogenesis and bone formation while simultaneously suppressing osteoclastic bone resorption through γ-carboxylation-independent mechanisms, such as the downregulation of NF-κB activation [69]

Disruptions to the gut microbiome, known as dysbiosis, can diminish the endogenous pool of vitamin K2, thereby impairing bone mineralisation and accelerating osteoporotic progression. Clinical evidence suggests that restoring these pathways—either through vitamin K2 supplementation or probiotic interventions such as *Lactobacillus rhamnosus*—can increase the cOC-to-ucOC ratio and preserve bone mineral density [70]. However, these findings need to be confirmed in randomised, placebo-controlled trials.

## 7. Dysbiosis and Bone Disease

Considering the data presented in the previous sections, the gut microbiome is increasingly recognised as an important regulator of bone mineral density through multiple, interrelated pathways. Experimental perturbations—such as faecal microbiota transplantation in germ-free mice and antibiotic exposure in conventionally raised animals—indicate that shifts in microbial community structure can materially influence bone turnover. In addition, microbiome acquisition and exchange (e.g., via maternal vertical transmission and cohabitation) can modify bone remodelling, supporting the concept that microbial ecology is a modifiable determinant of skeletal physiology [6].

Disruption of the gut microbiome (dysbiosis) may accelerate bone loss by increasing intestinal permeability, promoting systemic inflammation, and impairing nutrient absorption [71]. Increased intestinal permeability is characterised by reduced expression or integrity of tight junction components. This “leaky gut” phenotype facilitates the translocation of microbes and microbial products into systemic circulation, thereby sustaining low-grade inflammation and enabling downstream effects in extraintestinal tissues, including bone. For example, circulating liposaccharide (LPS) can promote bone resorption by inducing pro-inflammatory mediators (including TNF-α, IL-1, and IL-6) and by enhancing osteoclastogenesis via upregulation of RANKL and related signalling pathways [72]. Within this immunologic framework, chronic inflammatory states are associated with disturbances in the Treg/Th17 balance and elevations in chemokines and cytokines that expand osteoclast precursor pools and intensify osteoclast differentiation [73]. It should be noted, however, that the links between dysbiosis and Treg/Th17 imbalance are primarily based on preclinical studies. For example, in mice, dysbiosis-induced elevation of zonulin levels degrades essential tight junction proteins, severely impairing gut barrier function and promoting migration of Th17 cells from the gut to other sites, such as joints, potentially contributing to the onset of osteoporosis [74]. However, supplementation with *Lactobacillus rhamnosus* supported epithelial integrity and modulated the Th17/Treg balance, alleviating estrogen-deficiency-induced osteoporosis in animals [75].

Finally, the pathologic microbiome interfaces with innate immune-sensing pathways that can affect skeletal remodelling. Pattern-recognition receptors—including epithelial Toll-like receptor 5 and cytosolic NOD1/NOD2—can trigger NF-κB-dependent inflammatory pathways that increase RANKL signalling, thereby favouring osteoclast differentiation and bone resorption [76].

Collectively, these observations align with the growing field of osteoimmunology, which conceptualises skeletal homeostasis as an emergent property of coordinated immune, microbial, and endocrine networks [6]. There is also a growing body of evidence from observational studies linking dysbiosis to an increased risk of osteoporosis, osteoarthritis, and rheumatic diseases, such as rheumatoid arthritis [77,78,79]. It should be borne in mind, however, that these are multifactorial conditions and dysbiosis may represent only a single factor in their pathogenesis.

## 8. Therapeutic Implications

Given the significant role of the gut microbiota in bone metabolism and the negative impact of dysbiosis, several microbiota-targeted interventions are being explored to preserve and restore bone health.

### 8.1. Prebiotics

Prebiotics, such as inulin and fructooligosaccharides (FOSs), serve as targeted metabolic substrates for beneficial gut bacteria, thereby enhancing calcium absorption and producing systemic metabolites (mainly SCFAs) that regulate bone remodelling [80]. Prebiotics optimise the intestinal environment to maximise mineral bioavailability, a crucial factor when managing skeletal health alongside metabolic diseases. As described above, microbial short-chain fatty acids enhance colonic calcium absorption, support expansion of the absorptive mucosal surface, and upregulate transcellular transport proteins [16]. Moreover, prebiotics strengthen the tight junctions of the intestinal epithelial barrier, effectively reducing the translocation of bacterial endotoxins into the bloodstream. By dampening systemic inflammation, prebiotic supplementation may protect against the inflammatory bone loss typically observed in postmenopausal osteoporosis and metabolic syndrome, as shown in a recent meta-analysis of preclinical and clinical trials [81].

The mechanisms by which prebiotics and probiotics may influence calcium absorption and bone metabolism are summarised in Table 5 [82]. It should be noted, however, that most of the mechanisms and interactions described here are based on preclinical studies and will require verification in clinical settings.

### 8.2. Probiotics

Probiotics may influence bone metabolism by modulating systemic inflammation, endocrine pathways, and nutrient absorption, thereby supporting skeletal health. By restoring the gut microbiota, specific probiotic strains can inhibit osteoclast-mediated bone resorption and stimulate osteoblast activity, offering a promising adjunct approach for managing metabolic bone diseases. Beneficial mechanisms of probiotic action on bone metabolism are consistent with those of prebiotics in many respects (Table 5). In addition, the influence of probiotics on the Treg/T17 balance is important for suppressing inflammatory cytokine production and inhibiting the RANKL pathway [83]. It should be noted that most of the mechanisms described in Table 5 have been demonstrated primarily in animal models and therefore require verification in clinical trials.

In the few clinical trials conducted, probiotic interventions significantly reduce markers of bone turnover, such as bone-specific alkaline phosphatase (BALP), while stabilising serum calcium and PTH levels [80]. The most extensively studied strains belong to the Lactobacillus and Bifidobacterium genera (Table 6) [82,83,84,85,86]. A major limitation in the clinical use of probiotics is the considerable methodological heterogeneity and differences in study group sizes. Nevertheless, a recent meta-analysis suggests that supplementation with specific probiotic strains significantly improves bone mineral density, bone volume, and trabecular number in vivo [87]. These interventions might be helpful in reversing estrogen-deficiency-induced osteoporosis and inflammation-linked bone degradation by restoring microbial balance. As a result, selected probiotic strains may represent a promising therapeutic avenue for treating metabolic bone disorders and preventing osteoporosis in ageing populations.

### 8.3. Postbiotics

Postbiotics, formally defined as preparations of inanimate microorganisms and/or their components that confer a physiological benefit, encompass a diverse array of bioactive compounds, including cell-free supernatants, microbial lysates, and metabolic byproducts such as SCFAs. Preclinical data suggest that these compounds might modulate skeletal health by several mechanisms. By upregulating tight junction proteins (such as claudin-1), postbiotics reinforce the intestinal epithelial barrier, thereby mitigating increased intestinal permeability and indirectly dampening chronic, low-grade systemic inflammation—a primary driver of cytokine-mediated bone loss in degenerative skeletal diseases [88]. In addition, histomorphometric and biochemical studies suggest that specific postbiotic formulations can suppress osteoclastogenesis, resulting in reduced bone resorption markers, such as the C-terminal telopeptide of type I collagen. Simultaneously, these microbial derivatives promote osteoblast differentiation, increasing the prevalence of osteocalcin-positive osteoblasts and elevating serum concentrations of bone formation markers such as procollagen type 1 N-terminal propeptide [89].

In experimental models of metabolic and inflammatory osteopathies, such as postmenopausal and colitis-complicated osteoporosis, administration of specific postbiotics—such as *Bacillus* or *Lactobacillus* lysates—preserved trabecular bone microarchitecture, improved total bone volume, and significantly increased bone mineral density [90]. Since postbiotics eliminate the risk of opportunistic bacteremia and offer greater stability than live probiotics, they might serve as an adjunctive strategy in the clinical management of structural skeletal deterioration. The limited number of well-designed randomised trials involving postbiotics remains a barrier to their wider use in the prevention and treatment of bone diseases. Nevertheless, several studies are currently underway, for example, on the effect of butyrate on the clinical course and bone mineral density in patients with rheumatoid arthritis [91].

## 9. Conclusions

The gut microbiota functions as a powerful modulator of skeletal health—influencing calcium uptake, bone cell differentiation, hormonal signalling, and immune regulation in a tightly interconnected network. Under physiological conditions, the gut microbiota plays a crucial role in maintaining normal bone metabolism; however, microbial dysbiosis can adversely affect skeletal homeostasis. Consequently, microbiota-targeted interventions present a promising therapeutic strategy for both the prophylaxis and management of metabolic bone diseases like osteoporosis.

However, despite the growing recognition of the gut–bone axis, our understanding of how the gut microbiota impacts bone metabolism is constrained by methodological challenges and a reliance on correlational data. Advancing this field requires addressing critical research gaps before microbiome-targeted skeletal therapies can be reliably adopted in clinical settings. Firstly, most existing research relies on observational data, making it difficult to definitively establish causal mechanisms linking specific microbial profiles to bone remodelling. Next, amplicon-based analytical methods often lack the resolution to distinguish strain-specific metabolic pathways, and faecal metabolite measurements fail to accurately reflect true intestinal synthesis or systemic availability. Finally, high individual variability in gut flora and a lack of large-scale human clinical trials complicate the development of generalised, long-term therapeutic interventions like targeted probiotics or faecal microbiota transplantation.

Therefore, current research prospects focus on identifying specific beneficial microbial strains and elucidating how their metabolites modulate bone-resorbing osteoclasts and bone-forming osteoblasts via the immune system. To translate these molecular findings into clinical practice, future studies aim to leverage metagenomics to optimise the delivery and efficacy of targeted interventions, which include specialised probiotics, prebiotics, engineered microbes, and faecal microbiota transplantation. Ultimately, refining these microbiome-targeted strategies could provide personalised, non-pharmacological avenues for restoring microbial homeostasis and preserving bone mass in vulnerable populations.

## Figures and Tables

**Table 5:** Pathways of prebiotic and probiotic action on bone metabolism.

Target	Prebiotic Influence	Skeletal Outcome
Osteoclasts	Increased SCFAs synthesis suppress cellular differentiation and functional activity [37]	Decreased bone resorption and turnover
Osteoblasts	Increased SCFAs synthesis activates receptors to stimulate cellular proliferation [35,36]	Increased bone formation and mineralization
	Increased indole-3-propionic acid promotes mitochondrial biogenesis in osteoblasts [46,55]	
Intestinal Epithelium	Lower pH releases bound minerals and physically expand mucosal surface area [12,13,14]	Enhanced calcium bioavailability and total absorption
Immune System	Improved epithelial barrier function limits endotoxin translocation [18]	Prevention of systemic inflammatory bone loss

SCFAs—short-chain fatty acids.

**Table 6:** Impact of the selected *Lactobacillus* and *Bifidobacterium* strains on bone metabolism [82,83,84,85,86].

*Lactobacillus* Strain	Primary Cellular Target	Key Mechanisms of Action
*L. rhamnosus* GG	Osteoclasts	Suppresses NF-κB and modulates Th17/Treg balance
*L. acidophilus*	Osteoclasts	Increases colonic butyrate production and limits gut permeability
*L. plantarum*	Osteoblasts	Upregulates BMP pathway expression
*L. paracasei*	Osteoblasts Osteoclasts	Promotes BMP pathway while simultaneously inhibiting RANKL
* B. longum *	Osteoblasts Osteoclasts	Enhances bFGF expression in the bone microenvironment Suppresses NF-κB and modulates Th17/Treg balance

bFGF—basic fibroblast growth factor; BMP—bone morphogenetic protein; NF-κB—nuclear factor κB; RANKL—receptor activator for nuclear factor κB ligand; Th17—T helper 17 cells, Treg—T regulatory Cells.

## Data Availability

Not applicable.

## Supplementary Materials

The following supporting information can be downloaded at: https://www.mdpi.com/article/10.3390/ijms27093865/s1.

## Abbreviations

AhR	aryl hydrocarbon receptor
AMPK	AMP activated protein kinase
bFGF	basic fibroblast growth factor
BMP	bone morphogenetic protein
CaSR	calcium-sensing receptor
CD	cluster of differentiation
CTLA-4	cytotoxic T-lymphocyte-Associated Protein 4
cOC	carboxylated osteocalcin
ERK	extracellular signal-regulated kinase
FGF23	fibroblast growth factor 23
FXR	farnesoid X receptor
GM-CSF	granulocyte-macrophage colony-stimulating factor
GPCRs	G protein-coupled receptors
HDAC	histone deacetylase
HDCA	hyodeoxycholic acid
IFN	interferon
IGF-1	insulin-like growth factor 1
IGFBPs	insulin-like growth factor 1 binding proteins
IL	interleukin
IAA	indole-3-acetic acid
LPS	liposaccharide
NF-κB	nuclear factor κB
NOD	Nucleotide-binding oligomerization domain
PTH	parathormone
RANKL	receptor activator for nuclear factor κB ligand
RORγt	receptor gamma t
SCFAs	short-chain fatty acids
Th17	T helper 17 cells
TGR5	membrane G protein-coupled receptor
TGF β1	transforming growth factor β1
TNF-α	tumour necrosis factor α
Treg	regulatory T cells
TRPV6	transient receptor potential vanilloid 6 channel
ucOC	uncarboxylated osteocalcin
VDR	vitamin D receptor
Wnt10b	wingless-type MMTV integration site family, member 10B

## References

[1] Hou K., Wu Z.X., Chen X.Y., Wang J.Q., Zhang D., Xiao C., Zhu D., Koya J.B., Wei L., Li J. (2022). Microbiota in health and diseases. Signal Transduct. Target. Ther..

[2] Mukhopadhya I., Louis P. (2025). Gut microbiota-derived short-chain fatty acids and their role in human health and disease. Nat. Rev. Microbiol..

[3] Shen Y., Fan N., Ma S.X., Cheng X., Yang X., Wang G. (2025). Gut Microbiota Dysbiosis: Pathogenesis, Diseases, Prevention, and Therapy. MedComm.

[4] Yao D., Huang L., Ke J., Zhang M., Xiao Q., Zhu X. (2020). Bone Metabolism Regulation: Implications for the Treatment of Bone Diseases. Biomed. Pharmacother..

[5] Bolamperti S., Villa I., Rubinacci A. (2022). Bone remodelling: An operational process ensuring survival and bone mechanical competence. Bone Res..

[6] Indrio F., Salatto A. (2025). Gut Microbiota-Bone Axis. Ann. Nutr. Metab..

[7] Zhou Y., Yang Y., Zhu W., Kourkoumelis N., Wang Y., Chen Y., Hong L., Wang J., Zhu J., Zhu C. (2025). Microbial Influences on Calcium-Phosphorus Homeostasis and Metabolic Bone Diseases: A Bidirectional Mendelian Randomisation Study on the Gut-Bone Axis. J.Cell Mol. Med..

[8] Resch H., Zendeli A., Kocijan R. (2022). Metabolic Bone Diseases—A Topic of Great Diversity. J. Clin. Med..

[9] Baim S., Blank R. (2021). Approaches to Fracture Risk Assessment and Prevention. Curr. Osteoporos. Rep..

[10] Wang F., Wei W., Liu P.J. (2024). Effects of probiotic supplementation on bone health in postmenopausal women: A systematic review and meta-analysis. Front. Endocrinol..

[11] Raveschot C., Coutte F., Frémont M., Vaeremans M., Dugersuren J., Demberel S., Drider D., Dhulster P., Flahaut C., Cudennec B. (2020). Probiotic Lactobacillus strains from Mongolia improve calcium transport and uptake by intestinal cells in vitro. Food Res. Int..

[12] Thammayon N., Wongdee K., Teerapornpuntakit J., Panmanee J., Chanpaisaeng K., Charoensetakul N., Srimongkolpithak N., Suntornsaratoon P., Charoenphandhu N. (2024). Enhancement of intestinal calcium transport by short-chain fatty acids: Roles of Na^+^/H^+^ exchanger 3 and transient receptor potential vanilloid subfamily 6. Am. J. Physiol. Cell Physiol..

[13] Mineo H., Hara H., Tomita F. (2001). Short-chain fatty acids enhance diffusional ca transport in the epithelium of the rat cecum and colon. Life Sci..

[14] Nugent S.G., Kumar D., Rampton D.S., Evans D.F. (2001). Intestinal luminal pH in inflammatory bowel disease: Possible determinants and implications for therapy with aminosalicylates and other drugs. Gut.

[15] Kien C.L., Blauwiekel R., Bunn J.Y., Jetton T.L., Frankel W.L., Holst J.J. (2007). Cecal infusion of butyrate increases intestinal cell proliferation in piglets. J. Nutr..

[16] Parada Venegas D., De la Fuente M.K., Landskron G., González M.J., Quera R., Dijkstra G., Harmsen H.J.M., Faber K.N., Hermoso M.A. (2019). Short Chain Fatty Acids (SCFAs)-Mediated Gut Epithelial and Immune Regulation and Its Relevance for Inflammatory Bowel Diseases. Front. Immunol..

[17] Whisner C.M., Martin B.R., Schoterman M.H., Nakatsu C.H., McCabe L.D., McCabe G.P., Wastney M.E., van den Heuvel E.G., Weaver C.M. (2013). Galacto-oligosaccharides increase calcium absorption and gut bifidobacteria in young girls: A double-blind crossover trial. Br. J. Nutr..

[18] Peng L., Li Z.R., Green R.S., Holzman I.R., Lin J. (2009). Butyrate enhances the intestinal barrier by facilitating tight-junction assembly via AMP-activated protein kinase activation in Caco-2 cell monolayers. J. Nutr..

[19] Song P., Zhang X., Feng W., Xu W., Wu C., Xie S., Yu S., Fu R. (2023). Biological synthesis of ursodeoxycholic acid. Front. Microbiol..

[20] Hashimoto N., Matsui I., Ishizuka S., Inoue K., Matsumoto A., Shimada K., Hori S., Lee D.G., Yasuda S., Katsuma Y. (2020). Lithocholic acid increases intestinal phosphate and calcium absorption in a vitamin D receptor dependent but transcellular pathway independent manner. Kidney Int..

[21] Taparia S., Fleet J.C., Peng J.B., Wang X.D., Wood R.J. (2006). 1,25-Dihydroxyvitamin D and 25-hydroxyvitamin D—mediated regulation of TRPV6 (a putative epithelial calcium channel) mRNA expression in Caco-2 cells. Eur. J. Nutr..

[22] Collins S.L., Stine J.G., Bisanz J.E., Okafor C.D., Patterson A.D. (2023). Bile acids and the gut microbiota: Metabolic interactions and impacts on disease. Nat. Rev. Microbiol..

[23] Xiang T., Deng Z., Yang C., Tan J., Dou C., Luo F., Chen Y. (2023). Bile acid metabolism regulatory network orchestrates bone homeostasis. Pharmacol. Res..

[24] Ma H., Wang K., Jiang C. (2025). Microbiota-derived bile acid metabolic enzymes and their impacts on host health. Cell Insight.

[25] Fujimori K., Iguchi Y., Yamashita Y., Gohda K., Teno N. (2024). FXR Activation Accelerates Early Phase of Osteoblast Differentiation Through COX-2-PGE_2_-EP4 Axis in BMP-2-Induced Mouse Mesenchymal Stem Cells. Molecules.

[26] Cho S.W., An J.H., Park H., Yang J.Y., Choi H.J., Kim S.W., Park Y.J., Kim S.Y., Yim M., Baek W.Y. (2013). Positive regulation of osteogenesis by bile acid through FXR. J. Bone Miner. Res..

[27] Li Y., Hong Y., Shen H., Zhou J., Cesar D., Eleutério J., Matsuura M., Liu Y., Luo C., Li Q. (2025). FXR activation suppresses NF-κB signaling, proliferation and migration in cervical cancer cells. Transl. Cancer Res..

[28] Abu-Amer Y. (2013). NF-κB signaling and bone resorption. Osteoporos. Int..

[29] Zheng X.Q., Huang J., Yuan W.Q., Wu T., Wang H., Liu H., Zhang Y.D., He J.W., Huang C., Song C.L. (2025). Gut microbiota preserves bone mass through modulating the hyodeoxycholic acid-TGR5 axis. Gut Microbes.

[30] Priyadarshini M., Kotlo K.U., Dudeja P.K., Layden B.T. (2018). Role of Short Chain Fatty Acid Receptors in Intestinal Physiology and Pathophysiology. Compr. Physiol..

[31] You X., Huang D., Zhang Y., Zboinski E., Hodys C., Tsang K., Charles J.F. (2025). SCFA-GPR43 Axis Mediates Gut Microbiota Promotion of Juvenile Bone Growth. Curr. Dev. Nutr..

[32] Behler-Janbeck F., Baranowsky A., Yorgan T.A., Jaeckstein M.Y., Worthmann A., Fuh M.M., Gunasekaran K., Tiegs G., Amling M., Schinke T. (2024). The short-chain fatty acid receptors Gpr41/43 regulate bone mass by promoting adipogenic differentiation of mesenchymal stem cells. Front. Endocrinol..

[33] Chen J.R., Zhao H., Wankhade U.D., Chintapalli S.V., Li C., Gai D., Shankar K., Zhan F., Lazarenko O.P. (2021). GPR109A mediates the effects of hippuric acid on regulating osteoclastogenesis and bone resorption in mice. Commun. Biol..

[34] Fellows R., Denizot J., Stellato C., Cuomo A., Jain P., Stoyanova E., Balázsi S., Hajnády Z., Liebert A., Kazakevych J. (2018). Microbiota-derived short-chain fatty acids promote histone crotonylation in the colon through histone deacetylases. Nat. Commun..

[35] Schroeder T.M., Westendorf J.J. (2005). Histone deacetylase inhibitors promote osteoblast maturation. J. Bone Miner. Res..

[36] Fan X., Li L., Ye Z., Zhou Y., Tan W.S. (2018). Regulation of osteogenesis of human amniotic mesenchymal stem cells by sodium butyrate. Cell Biol. Int..

[37] Rahman M.M., Kukita A., Kukita T., Shobuike T., Nakamura T., Kohashi O. (2003). Two histone deacetylase inhibitors, trichostatin A and sodium butyrate, suppress differentiation into osteoclasts but not into macrophages. Blood.

[38] Rashed F., Kamijyo S., Shimizu Y., Hirohashi Y., Khan M., Sugamori Y., Murali R., Aoki K. (2021). The Effects of Receptor Activator of NF-κB Ligand-Binding Peptides on Bone Resorption and Bone Formation. Front. Cell Dev. Biol..

[39] Yan J., Charles J.F. (2018). Gut Microbiota and IGF-1. Calcif. Tissue Int..

[40] Wang Y., Nishida S., Elalieh H.Z., Long R.K., Halloran B.P., Bikle D.D. (2006). Role of IGF-I signaling in regulating osteoclastogenesis. J. Bone Miner. Res..

[41] Guan Z., Luo L., Liu S., Guan Z., Zhang Q., Li X., Tao K. (2022). The Role of Depletion of Gut Microbiota in Osteoporosis and Osteoarthritis: A Narrative Review. Front. Endocrinol..

[42] Schluter J., Peled J.U., Taylor B.P., Markey K.A., Smith M., Taur Y., Niehus R., Staffas A., Dai A., Fontana E. (2020). The gut microbiota is associated with immune cell dynamics in humans. Nature.

[43] Ono T., Takayanagi H. (2017). Osteoimmunology in Bone Fracture Healing. Curr. Osteoporos. Rep..

[44] Okamoto K., Nakashima T., Shinohara M., Negishi-Koga T., Komatsu N., Terashima A., Sawa S., Nitta T., Takayanagi H. (2017). Osteoimmunology: The conceptual framework unifying the immune and skeletal systems. Physiol. Rev..

[45] Saadh M.J., Allela O.Q.B., Ballal S., Mahdi M.S., Chahar M., Verma R., Al-Hussein R.K.A., Adil M., Jawad M.J., Al-Nuaimi A.M.A. (2025). The effects of microbiota-derived short-chain fatty acids on T lymphocytes: From autoimmune diseases to cancer. Semin. Oncol..

[46] Tyagi A.M., Yu M., Darby T.M., Vaccaro C., Li J.Y., Owens J.A., Hsu E., Adams J., Weitzmann M.N., Jones R.M. (2018). The Microbial Metabolite Butyrate Stimulates Bone Formation via T Regulatory Cell-Mediated Regulation of WNT10B Expression. Immunity.

[47] Wang J., Zhu N., Su X., Gao Y., Yang R. (2023). Gut-Microbiota-Derived Metabolites Maintain Gut and Systemic Immune Homeostasis. Cells.

[48] Baranwal G., Goodlett B.L., Arenaz C.M., Creed H.A., Navaneethabalakrishnan S., Rutkowski J.M., Alaniz R.C., Mitchell B.M. (2023). Indole Propionic Acid Increases T Regulatory Cells and Decreases T Helper 17 Cells and Blood Pressure in Mice with Salt-Sensitive Hypertension. Int. J. Mol. Sci..

[49] Hang S., Paik D., Yao L., Kim E., Trinath J., Lu J., Ha S., Nelson B.N., Kelly S.P., Wu L. (2019). Bile acid metabolites control T_H_17 and T_reg_ cell differentiation. Nature.

[50] Park E., Ciofani M. (2025). Th17 cell pathogenicity in autoimmune disease. Exp. Mol. Med..

[51] Kibbie J.J., Dillon S.M., Thompson T.A., Purba C.M., McCarter M.D., Wilson C.C. (2021). Butyrate directly decreases human gut lamina propria CD4 T cell function through histone deacetylase (HDAC) inhibition and GPR43 signalling. Immunobiology.

[52] Miyako K., Kanno T., Endo T., Yoshida S., Iwao Y., Nakano K., Ito A., Yokoyama S., Asou H.K., Yamada K. (2025). The RORγt ligand-binding domain controls the pathogenicity of IL-17A^+^ T cells differently in autoimmune diseases of the skin and CNS. Cell Rep..

[53] Park J., Kim M., Kang S.G., Jannasch A.H., Cooper B., Patterson J., Kim C.H. (2015). Short-chain fatty acids induce both effector and regulatory T cells by suppression of histone deacetylases and regulation of the mTOR-S6K pathway. Mucosal Immunol..

[54] Luu M., Pautz S., Kohl V., Singh R., Romero R., Lucas S., Hofmann J., Raifer H., Vachharajani N., Carrascosa L.C. (2019). The short-chain fatty acid pentanoate suppresses autoimmunity by modulating the metabolic-epigenetic crosstalk in lymphocytes. Nat. Commun..

[55] Kim C.H. (2021). Control of lymphocyte functions by gut microbiota-derived short-chain fatty acids. Cell Mol. Immunol..

[56] Chen B., Ye D., Luo L., Liu W., Peng K., Shu X., Gu W., Wang X., Xiang C., Jiang M. (2020). Adhesive bacteria in the terminal ileum of children correlates with increasing Th17 cell activation. Front. Pharmacol..

[57] Sun S., Luo L., Liang W., Yin Q., Guo J., Rush A.M., Lv Z., Liang Q., Fischbach M.A., Sonnenburg J.L. (2020). Bifidobacterium alters the gut microbiota and modulates the functional metabolism of T regulatory cells in the context of immune checkpoint blockade. Proc. Natl. Acad. Sci. USA.

[58] Liu H., Yi P., Zhao W., Wu Y., Acher F., Pin J.P., Liu J., Rondard P. (2020). lluminating the allosteric modulation of the calcium-sensing receptor. Proc. Natl. Acad. Sci. USA.

[59] Sun Y., Song J., Liu H., Li L., Xiao K., Mao W., Jiang C. (2023). Calcium-sensing receptor alleviates gut damage caused by endotoxemia by regulating the gut microbiota. Transl. Pediatr..

[60] D’Amelio P., Sassi F., Buondonno I., Fornelli G., Spertino E., D’Amico L., Marchetti M., Lucchiari M., Roato I., Isaia G.C. (2015). Treatment with intermittent PTH increases Wnt10b production by T cells in osteoporotic patients. Osteoporos. Int..

[61] Tangestani H., Boroujeni H.K., Djafarian K., Emamat H., Shab-Bidar S. (2021). Vitamin D and The Gut Microbiota: A Narrative Literature Review. Clin. Nutr. Res..

[62] Sawada N., Sakaki T., Yoneda S., Kusudo T., Shinkyo R., Ohta M., Inouye K. (2004). Conversion of vitamin D3 to 1α,25-dihydroxyvitamin D3 by Streptomyces griseolus cytochrome P450SU-1. Biochem. Biophys. Res. Commun..

[63] Razzaque M.S. (2022). Interactions between FGF23 and vitamin D. Endocr. Connect..

[64] Soto-Martin E.C., Warnke I., Farquharson F.M., Christodoulou M., Horgan G., Derrien M., Faurie J.M., Flint H.J., Duncan S.H., Louis P. (2020). Vitamin Biosynthesis by Human Gut Butyrate-Producing Bacteria and Cross-Feeding in Synthetic Microbial Communities. mBio.

[65] Sun J., Zhang Y.G. (2022). Vitamin D Receptor Influences Intestinal Barriers in Health and Disease. Cells.

[66] Keyvan E., Adesemoye E., Champomier-Vergès M.C., Chanséaume-Bussiere E., Mardon J., Nedelkoska D.N., Palamutoglu R., Russo P., Sarand I., Songre-Ouattara L. (2025). Vitamins formed by microorganisms in fermented foods: Effects on human vitamin status—A systematic narrative review. Front. Nutr..

[67] Mandatori D., Pelusi L., Schiavone V., Pipino C., Di Pietro N., Pandolfi A. (2021). The Dual Role of Vitamin K2 in “Bone-Vascular Crosstalk”: Opposite Effects on Bone Loss and Vascular Calcification. Nutrients.

[68] Gundberg C.M., Lian J.B., Booth S.L. (2012). Vitamin K-dependent carboxylation of osteocalcin: Friend or foe?. Adv. Nutr..

[69] Yamaguchi M., Weitzmann M.N. (2011). Vitamin K2 stimulates osteoblastogenesis and suppresses osteoclastogenesis by suppressing NF-κB activation. Int. J. Mol. Med..

[70] Xie C., Gong J., Zheng C., Zhang J., Gao J., Tian C., Guo X., Dai S., Gao T. (2024). Effects of vitamin K supplementation on bone mineral density at different sites and bone metabolism in the middle-aged and elderly population. Bone Jt. Res..

[71] Schepper J.D., Collins F.L., Rios-Arce N.D., Raehtz S., Schaefer L., Gardinier J.D., Britton R.A., Parameswaran N., McCabe L.R. (2019). Probiotic Lactobacillus reuteri Prevents Postantibiotic Bone Loss by Reducing Intestinal Dysbiosis and Preventing Barrier Disruption. J. Bone Miner. Res..

[72] Ulluwishewa D., Anderson R.C., McNabb W.C., Moughan P.J., Wells J.M., Roy N.C. (2011). Regulation of tight junction permeability by intestinal bacteria and dietary components. J. Nutr..

[73] Su X., Yin X., Liu Y., Yan X., Zhang S., Wang X., Lin Z., Zhou X., Gao J., Wang Z. (2020). Gut Dysbiosis Contributes to the Imbalance of Treg and Th17 Cells in Graves’ Disease Patients by Propionic Acid. J. Clin. Endocrinol. Metab..

[74] Qi P., Xie R., Liu H., Zhang Z., Cheng Y., Ma J., Wan K., Xie X. (2024). Mechanisms of gut homeostasis regulating Th17/Treg cell balance in PMOP. Front. Immunol..

[75] Locantore P., Del Gatto V., Gelli S., Paragliola R.M., Pontecorvi A. (2020). The interplay between immune system and microbiota in osteoporosis. Mediat. Inflamm..

[76] Kishimoto T., Kaneko T., Ukai T., Yokoyama M., Ayon Haro R., Yoshinaga Y., Yoshimura A., Hara Y. (2012). Peptidoglycan and lipopolysaccharide synergistically enhance bone resorption and osteoclastogenesis. J. Periodontal Res..

[77] Lai J., Gong L., Liu Y., Zhang X., Liu W., Han M., Zhou D., Shi S. (2024). Associations between gut microbiota and osteoporosis or osteopenia in a cohort of Chinese Han youth. Sci. Rep..

[78] Liu S., Li G., Xu H., Wang Q., Wei Y., Yang Q., Xiong A., Yu F., Weng J., Zeng H. (2023). “Cross-talk” between gut microbiome dysbiosis and osteoarthritis progression: A systematic review. Front. Immunol..

[79] Cheng M., Zhao Y., Cui Y., Zhong C., Zha Y., Li S., Cao G., Li M., Zhang L., Ning K. (2022). Stage-specific roles of microbial dysbiosis and metabolic disorders in rheumatoid arthritis. Ann. Rheum. Dis..

[80] Whisner C.M., Castillo L.F. (2018). Prebiotics, Bone and Mineral Metabolism. Calcif. Tissue Int..

[81] Ghorbani Z., Shoaibinobarian N., Noormohammadi M., Taylor K., Kazemi A., Bonyad A., Khoshdooz S., Löber U., Forslund-Startceva S.K. (2025). Reinforcing gut integrity: A systematic review and meta-analysis of clinical trials assessing probiotics, synbiotics, and prebiotics on intestinal permeability markers. Pharmacol. Res..

[82] Fu J., Jia L., Wu L., Jiang Y., Zhao R., Du J., Guo L., Zhang C., Xu J., Liu Y. (2024). Lactobacillus rhamnosus inhibits osteoclast differentiation by suppressing the TLR2/NF-κB pathway. Oral. Dis..

[83] Dong J., Shu G., Yang J., Wang B., Chen L., Gong Z., Zhang X. (2024). Mechanistic study on the alleviation of postmenopausal osteoporosis by Lactobacillus acidophilus through butyrate-mediated inhibition of osteoclast activity. Sci. Rep..

[84] Yang L.C., Lin S.W., Li I.C., Chen Y.P., Tzu S.Y., Chou W., Chen C.C., Lin W.C., Chen Y.L., Lin W.H. (2020). *Lactobacillus* plantarum GKM3 and Lactobacillus paracasei GKS6 Supplementation Ameliorates Bone Loss in Ovariectomized Mice by Promoting Osteoblast Differentiation and Inhibiting Osteoclast Formation. Nutrients.

[85] Wu Y., Yang Y., Wang L., Chen Y., Han X., Sun L., Chen H., Chen Q. (2023). Effect of Bifidobacterium on osteoclasts: TNF-α/NF-κB inflammatory signal pathway-mediated mechanism. Front. Endocrinol..

[86] Triwardhani A., Anggitia C., Ardani I.G.A.W., Nugraha A.P., Riawan W. (2021). The increased basic fibroblast growth factor expression and osteoblast number post *Bifidobacterium bifidum* probiotic supplementation during orthodontic tooth movement in Wistar rats. J. Pharm. Pharmacogn. Res..

[87] Harini J.N., Gayarthi G., Mahadevan S., Ilangovan R. (2025). Effects of probiotic supplements on bone mineral density and bone turnover markers in postmenopausal women: A systematic review. Clin. Nutr. ESPEN.

[88] Yu T., Bai R., Wang Z., Qin Y., Wang J., Wei Y., Zhao R., Nie G., Han B. (2024). Colon-targeted engineered postbiotics nanoparticles alleviate osteoporosis through the gut-bone axis. Nat. Commun..

[89] Yadav S., Sapra L., Srivastava R.K. (2024). Polysaccharides to postbiotics: Nurturing bone health via modulating “gut-immune axis”. Int. J. Biol. Macromol..

[90] Lim Y., Park O.J., Park C., Kim B.M., Yun C.H., Han S.H. (2026). Oral intake of Heat-Killed *Lactiplantibacillus plantarum* Alleviates Bone Loss in an Ovariectomized Mouse Model Similarly to Live *L. plantarum*. J. Microbiol. Biotechnol..

[91] https://clinicaltrials.gov/study/NCT05718583?tab=study.

