# Supplementary material for: Impact of Gut Microbiota on Bone Metabolism—Present Concepts and Therapeutic Implications
Source: Int J Mol Sci. 2026 Apr 27;27(9):3865. doi: 10.3390/ijms27093865 (PMC13163506; doi:10.3390/ijms27093865)
Supplement: Supplementary file 1 [file ijms-27-03865-s001.zip › ijms-4204743-supplementary.pdf]

Supplementary Table S1. Publications included in the mini-review, considering the research models used/publication type.

Publication	
**Research articles**	**Research model**
Baranwal, G.; Goodlett, B. L.; Arenaz, C. M.; Creed, H. A.; Navaneethabalakrishnan, S.; Rutkowski, J. M.; Alaniz, R. C.; Mitchell, B. M. Indole Propionic Acid Increases T Regulatory Cells and Decreases T Helper 17 Cells and Blood Pressure in Mice with Salt-Sensitive Hypertension. *Int J Mol Sci*. 2023;24(11):9192. Published 2023 May 24. DOI: 10.3390/ijms24119192	Animal study/postbiotics
Behler-Janbeck, F.; Baranowsky, A.; Yorgan, T. A.; Jaeckstein, M. Y.; Worthmann, A.; Fuh, M. M.; Gunasekaran, K.; Tiegs, G.; Amling, M.; Schinke, T.; Heeren, J. The short-chain fatty acid receptors Gpr41/43 regulate bone mass by promoting adipogenic differentiation of mesenchymal stem cells. *Front Endocrinol (Lausanne)* **2024**, *15*, 1392418. doi:10.3389/fendo.2024.1392418. Erratum in: Front Endocrinol (Lausanne). 2025 Jan 07;15:1528968. DOI: 10.3389/fendo.2024.1392418	In vitro
Chen, B.; Ye, D.; Luo, L.; Liu, W.; Peng, K.; Shu, X.; Gu, W.; Wang, X.; Xiang, C.; Jiang, M. Adhesive bacteria in the terminal ileum of children correlates with increasing Th17 cell activation. *Front Pharmacol* **2020**, *11*, 588560. doi: 10.3389/fphar.2020.588560	In vitro
Chen, J. R.; Zhao, H.; Wankhade, U. D.; Chintapalli, S. V.; Li, C.; Gai, D.; Shankar, K.; Zhan, F.; Lazarenko, O. P. GPR109A mediates the effects of hippuric acid on regulating osteoclastogenesis and bone resorption in mice. *Commun Biol* **2021**, *4*, 53. DOI: 10.1038/s42003-020-01564-2	In vitro/animal study/ postbiotics
Cho, S.W.; An, J. H.; Park, H.; Yang, J. Y.; Choi, H. J.; Kim, S. W.; Park, Y. J.; Kim, S. Y.; Yim, M.; Baek, W. Y.; Kim, J. E.; Shin, C. S. Positive regulation of osteogenesis by bile acid through FXR. *J Bone Miner Res* **2013**, *28*, 2109-2121. DOI: 10.1002/jbmr.1961	In vitro/animal study/ postbiotics
D'Amelio, P.; Sassi, F.; Buondonno, I.; Fornelli, G.; Spertino, E.; D'Amico, L.; Marchetti, M.; Lucchiari, M.; Roato, I.; Isaia, G. C. Treatment with intermittent PTH increases Wnt10b production by T cells in osteoporotic patients. *Osteoporos Int* **2015**, *26*, 2785–2791. DOI: 10.1007/s00198-015-3189-8	Clinical trial
Dong, J.; Shu, G.; Yang, J.; Wang, B.; Chen, L.; Gong, Z; Zhang, X. Mechanistic study on the alleviation of postmenopausal osteoporosis by Lactobacillus acidophilus through butyrate-mediated inhibition of osteoclast activity. *Sci Rep* **2024**, *14*, 7042. DOI: 10.1038/s41598-024-57122-x	Observational study in humans /Animal study/probiotics
Fan, X.; Li, L.; Ye, Z.; Zhou, Y.; Tan, W.S. Regulation of osteogenesis of human amniotic mesenchymal stem cells by sodium butyrate. *Cell Biol Int* **2018**, *42*, 457-469. DOI: 10.1002/cbin.10919	In vitro/ postbiotics
Fellows, R.; Denizot, J.; Stellato, C.; Cuomo, A.; Jain, P.; Stoyanova, E.; Balázsi, S.; Hajnády, Z.; Liebert, A.; Kazakevych, J.; Blackburn, H.; Corrêa, R. O.; Fachi, J. L.; Sato, F. T.; Ribeiro, W. R.; Ferreira, C. M.; Perée, H.; Spagnuolo, M.; Mattiuz, R.; Matolcsi, C.;… Varga-Weisz, P. Microbiota-derived short-chain fatty acids promote histone crotonylation in the colon through histone deacetylases. *Nat Commun* **2018**, *9*, 105. DOI: 10.1038/s41467-017-02651-5	Animal study/ postbiotics
Fu, J.; Jia, L.; Wu, L.; Jiang, Y.; Zhao, R.; Du, J.; Guo, L.; Zhang, C.; Xu, J.; Liu, Y. Lactobacillus rhamnosus inhibits osteoclast differentiation by suppressing the TLR2/NF-κB pathway. *Oral dis* **2024**, *30*, 2373–2386. DOI: 10.1111/odi.14712	In vitro/probiotic
Fujimori, K.; Iguchi, Y.; Yamashita, Y.; Gohda, K.; Teno, N. FXR Activation Accelerates Early Phase of Osteoblast Differentiation Through COX-2-PGE_2-EP4 Axis in BMP-2-Induced Mouse Mesenchymal Stem Cells. *Molecules* 2024, 30, 58. doi:10.3390/molecules30010058	In vitro
H. K.; Yamada, K.; Kimura, T.; Nakayama, M.; Ohara, O.; Endo, Y. The RORγt ligand-binding domain controls the pathogenicity of IL-17A^{+} T cells differently in autoimmune diseases of the skin and CNS. *Cell Rep* **2025**, *44*, 116700. DOI: 10.3390/molecules30010058	Animal study

Hang, S.; Paik, D.; Yao, L.; Kim, E.; Trinath, J.; Lu, J.; Ha, S.; Nelson, B. N.; Kelly, S. P.; Wu, L.; Zheng, Y.; Longman, R. S.; Rastinejad, F.; Devlin, A. S.; Krout, M. R.; Fischbach, M. A.; Littman, D. R.; Huh, J. R. Bile acid metabolites control T_H17 and T_{reg} cell differentiation. *Nature*. 2019;576(7785):143-148. DOI: 10.1038/s41586-019-1785-z	In vitro/ postbiotics
Hashimoto, N., Matsui, I., Ishizuka, S., Inoue, K., Matsumoto, A., Shimada, K., Hori, S., Lee, D. G., Yasuda, S., Katsuma, Y., Kajimoto, S., Doi, Y., Yamaguchi, S., Kubota, K., Oka, T., Sakaguchi, Y., Takabatake, Y., Hamano, T., Isaka, Lithocholic acid increases intestinal phosphate and calcium absorption in a vitamin D receptor dependent but transcellular pathway independent manner. *Kidney Int* **2020**, *97*, 1164-1180. DOI: 10.1016/j.kint.2020.01.032	Animal study/postbiotics
Kibbie, J.J.; Dillon, S.M.; Thompson, T.A.; Purba, C.M.; McCarter, M.D.; Wilson, C.C. Butyrate directly decreases human gut lamina propria CD4 T cell function through histone deacetylase (HDAC) inhibition and GPR43 signalling. *Immunobiology* **2021**, *226*, 152126. DOI: 10.1016/j.imbio.2021.152126	In vitro/ postbiotics
Kien, C.L.; Blauwiekel, R.; Bunn, J.Y.; Jetton, T.L.; Frankel, W.L.; Holst, J.J. Cecal infusion of butyrate increases intestinal cell proliferation in piglets. *J. Nutr* **2007**, *137*, 916–922. DOI: 10.1093/jn/137.4.916	Animal study/postbiotics
Kim, Y. G.; Lee, C. K.; Nah, S. S.; Mun, S. H.; Yoo, B.; Moon, H. B. Human CD4+CD25+ regulatory T cells inhibit the differentiation of osteoclasts from peripheral blood mononuclear cells. *Biochem Biophys Res Commun* **2007**, *357*, 1046-1052. DOI: 10.1016/j.bbrc.2007.04.042	In vitro
Kishimoto, T.; Kaneko, T.; Ukai, T.; Yokoyama, M.; Ayon Haro, R.; Yoshinaga, Y.; Yoshimura, A.; Hara, Y. Peptidoglycan and lipopolysaccharide synergistically enhance bone resorption and osteoclastogenesis. J Periodontal Res 2012, 47, 446–454. DOI: 10.1111/j.1600-0765.2011.01452.x	Animal study/postbiotics
Lai, J.; Gong, L.; Liu, Y.; Zhang, X.; Liu, W.; Han, M.; Zhou, D.; Shi, S. Associations between gut microbiota and osteoporosis or osteopenia in a cohort of Chinese Han youth. *Sci Rep* **2024**, *14*, 20948. Published 2024 Sep 9. DOI: 10.1038/s41598-024-71731-6	Observational study in humans
Li, Y.; Hong, Y.; Shen, H.; Zhou, J.; Cesar, D.; Eleutério, J.Jr; Matsuura, M.; Liu, Y.; Luo, C.; Li, Q. FXR activation suppresses NF-κB signaling, proliferation and migration in cervical cancer cells. *Transl Cancer Res* **2025**, *14*, 2440-2456. DOI: 10.1038/s41598-024-71731-6	In vitro
Lim, Y.; Park, O.J.; Park, C.; Kim, B.M.; Yun, C.H.; Han, S.H. Oral intake of Heat-Killed *Lactiplantibacillus plantarum* Alleviates Bone Loss in an Ovariectomized Mouse Model Similarly to Live *L. plantarum*. *J Microbiol Biotechnol* 2026,36, e2510013. DOI: 10.4014/jmb.2510.10013	Animal study/postbiotics
Liu, H.; Yi, P.; Zhao, W.; Wu, Y.; Acher, F.; Pin, J. P.; Liu, J.; Rondard, P. llluminating the allosteric modulation of the calcium-sensing receptor. *Proc Natl Acad Sci U S A* **2020**, *117*, 21711-21722. DOI: 10.1073/pnas.1922231117	In vitro
Luu, M.; Pautz, S.; Kohl, V.; Singh, R.; Romero, R.; Lucas, S.; Hofmann, J.; Raifer, H.; Vachharajani, N.; Carrascosa, L. C.; Lamp, B.; Nist, A.; Stiewe, T.; Shaul, Y.; Adhikary, T.; Zaiss, M. M.; Lauth, M.; Steinhoff, U.; Visekruna, A. The short-chain fatty acid pentanoate suppresses autoimmunity by modulating the metabolic-epigenetic crosstalk in lymphocytes. *Nat Commun* **2019**, *10*, 760. DOI: 10.1038/s41467-019-08711-2	In vitro/animal study/ postbiotics
Mineo, H.; Hara, H., Tomita, F. Short-chain fatty acids enhance diffusional ca transport in the epithelium of the rat cecum and colon. *Life Sci* **2001**, *69*, 517-526. DOI: 10.1016/s0024-3205(01)01146-8	Animal study/postbiotics
Miyako, K.; Kanno, T.; Endo, T.; Yoshida, S.; Iwao, Y.; Nakano, K.; Ito, A.; Yokoyama, S.; Asou, Sun, S.; Luo, L.; Liang, W.; Yin, Q.; Guo, J.; Rush, A. M.; Lv, Z.; Liang, Q.; Fischbach, M. A.; Sonnenburg, J. L.; Dodd, D.; Davis, M. M.; Wang, F. Bifidobacterium alters the gut microbiota and modulates the functional metabolism of T regulatory cells in the context of immune checkpoint blockade. *Proc Natl Acad Sci U S A* **2020**, 117, 27509–27515. DOI: 10.1073/pnas.1921223117	In vitro/animal study/ probiotics

Park, J.; Kim, M.; Kang, S.G.; Jannasch, A.H.; Cooper, B.; Patterson, J.; Kim CH. Short-chain fatty acids induce both effector and regulatory T cells by suppression of histone deacetylases and regulation of the mTOR-S6K pathway. *Mucosal Immunol* **2015**, *8*, 80-93. DOI: 10.1038/mi.2014.44	In vitro/animal study/ postbiotics
Peng, L.; Li, Z. R.; Green, R. S.; Holzman, I. R.; Lin, J. Butyrate enhances the intestinal barrier by facilitating tight-junction assembly via AMP-activated protein kinase activation in Caco-2 cell monolayers. *J Nutr* 2009, *139*, 1619–1625. DOI: 10.3945/jn.109.104638	In vitro/postbiotics
Rahman, M.M.; Kukita, A.; Kukita, T.; Shobuike, T.; Nakamura, T.; Kohashi, O. Two histone deacetylase inhibitors, trichostatin A and sodium butyrate, suppress differentiation into osteoclasts but not into macrophages. *Blood* **2003**, *101*, 3451-345. DOI: 10.1182/blood-2002-08-2622	In vitro/ postbiotics
Raveschot, C.; Coutte, F.; Frémont, M.; Vaeremans, M.; Dugersuren, J.; Demberel, S.; Drider, D.; Dhulster, P.; Flahaut, C; Cudennec. Probiotic Lactobacillus strains from Mongolia improve calcium transport and uptake by intestinal cells in vitro. *Food Res Int* 2020, *133*, 109201. DOI: 10.1177/1747493018778713	In vitro/probiotics
Sato, K.; Suematsu, A.; Okamoto, K.; Yamaguchi, A.; Morishita, Y.; Kadono, Y.; Tanaka, S.; Kodama, T.; Akira, S.; Iwakura, Y.; Cua, D. J.; Takayanagi, H. Th17 functions as an osteoclastogenic helper T cell subset that links T cell activation and bone destruction. *J Exp Med* **2006**, *203*, 2673-2682. DOI: 10.1084/jem.20061775	In vitro/animal study
Sawada, N.; Sakaki, T.; Yoneda, S.; Kusudo, T.; Shinkyo, R.; Ohta, M.; Inouye, K. Conversion of vitamin D3 to 1alpha,25-dihydroxyvitamin D3 by Streptomyces griseolus cytochrome P450SU-1. *Biochem Biophys Res Commun* **2004**, *320*, 156–164. DOI: 10.1016/j.bbrc.2004.05.140	In vitro
Schepper, J. D.; Collins, F. L.; Rios-Arce, N. D.; Raehtz, S.; Schaefer, L.; Gardinier, J. D.; Britton, R. A.; Parameswaran, N.; McCabe, L. R. Probiotic Lactobacillus reuteri Prevents Postantibiotic Bone Loss by Reducing Intestinal Dysbiosis and Preventing Barrier Disruption. *J Bone Miner Res* **2019**, *34*, 681–698. DOI:org/10.1002/jbmr.3635	Animal study/probiotics
Schluter, J.; Peled, J. U.; Taylor, B. P.; Markey, K. A.; Smith, M.; Taur, Y.; Niehus, R.; Staffas, A.; Dai, A.; Fontana, E.; Amoretti, L. A.; Wright, R. J.; Morjaria, S.; Fenelus, M.; Pessin, M. S.; Chao, N. J.; Lew, M.; Bohannon, L.; Bush, A.; Sung, A. D.; … Xavier, J. B. The gut microbiota is associated with immune cell dynamics in humans. *Nature* **2020**, *588*, 303–307. DOI: 10.1038/s41586-020-2971-8	Analysis of high-resolution clinical metadata obtained from human whole blood cells and gut microbiota
Schroeder, T. M.; Westendorf, J. J. Histone deacetylase inhibitors promote osteoblast maturation. *J Bone Miner Res* **2005**, *20*, 2254–2263. DOI: 10.1359/JBMR.050813	In vitro/ postbiotics
Soto-Martin, E. C.; Warnke, I.; Farquharson, F. M.; Christodoulou, M.; Horgan, G.; Derrien, M.; Faurie, J. M.; Flint, H. J.; Duncan, S. H.; Louis, P. Vitamin Biosynthesis by Human Gut Butyrate-Producing Bacteria and Cross-Feeding in Synthetic Microbial Communities. *mBio* **2020**, *11*, e00886-20. DOI: 10.1128/mBio.00886-20	In vitro
Su, X.; Yin, X.; Liu, Y.; Yan, X.; Zhang, S.; Wang, X.; Lin, Z.; Zhou, X.; Gao, J.; Wang, Z.; Zhang, Q. Gut Dysbiosis Contributes to the Imbalance of Treg and Th17 Cells in Graves' Disease Patients by Propionic Acid. *J Clin Endocrinol Metab* **2020**, *105*, dgaa511. DOI: 10.1210/clinem/dgaa511	Observational study in humans
Sun, Y.; Song, J.; Liu, H.; Li, L.; Xiao, K.; Mao, W.; Jiang, C. Calcium-sensing receptor alleviates gut damage caused by endotoxemia by regulating the gut microbiota. T*ransl Pediatr* **2023**, *12*, 2179–2190. DOI: 10.21037/tp-23-327	Animal study
Taparia, S.; Fleet, J.C.; Peng, J.B.; Wang, X.D.; Wood, R.J. 1,25-Dihydroxyvitamin D and 25-hydroxyvitamin D--mediated regulation of TRPV6 (a putative epithelial calcium channel) mRNA expression in Caco-2 cells. *Eur J Nutr* **2006**, *45*, 196-204. DOI: 10.1007/s00394-005-0586-3	In vitro
Thammayon, N.; Wongdee, K.; Teerapornpuntakit, J.; Panmanee, J.;Chanpaisaeng, K.; Charoensetakul, N.; Srimongkolpithak, N.; Suntornsaratoon, P.; Charoenphandhu, N. Enhancement of intestinal calcium	Animal study/postbiotics

transport by short-chain fatty acids: roles of Na+/H+ exchanger 3 and transient receptor potential vanilloid subfamily 6. *Am J Physiol Cell Physiol* **2024**, *326*, C317–C330. DOI: 10.1152/ajpcell.00330.2023	
Triwardhani, A.; Anggitia, C.; Ardani, I.G.A.W.; Nugraha, A.P.; Riawan, W(2021). The increased basic fibroblast growth factor expression and osteoblast number post *Bifidobacterium bifidum* probiotic supplementation during orthodontic tooth movement in Wistar rats. *J Pharm Pharmacogn Res* **2021**, *9*, 446–453. DOI: https://doi.org/10.56499/jppres21.1010_9.4.446	Animal study/probiotics
Tyagi, A. M.; Yu, M.; Darby, T. M.; Vaccaro, C.; Li, J. Y.; Owens, J. A.; Hsu, E.; Adams, J.; Weitzmann, M. N.; Jones, R. M.; Pacifici, R. The Microbial Metabolite Butyrate Stimulates Bone Formation via T Regulatory Cell-Mediated Regulation of WNT10B Expression. *Immunity* **2018**, *49*, 1116-1131.e7. DOI: 10.1016/j.immuni.2018.10.013	In vitro/ postbiotics
Whisner, C. M.; Martin, B. R.; Schoterman, M. H.; Nakatsu, C. H.; McCabe, L. D.; McCabe, G. P.; Wastney, M. E.; van den Heuvel, E. G.; Weaver, C. M. Galacto-oligosaccharides increase calcium absorption and gut bifidobacteria in young girls: a double-blind crossover trial. *Br J Nutr* **2013**, *110*,1292–303. DOI: 10.1017/S000711451300055X	Clinical trial/prebiotics
Xie, C.; Gong, J.; Zheng, C.; Zhang, J.; Gao, J.; Tian, C.; Guo, X.; Dai, S.; Gao, T. Effects of vitamin K supplementation on bone mineral density at different sites and bone metabolism in the middle-aged and elderly population. *Bone Joint Res* **2024**, *13*, 750-763. DOI: 10.1302/2046-3758.1312.BJR-2024-0053.R1	Clinical trail
Yamaguchi, M.; Weitzmann, M. N. Vitamin K2 stimulates osteoblastogenesis and suppresses osteoclastogenesis by suppressing NF-κB activation. *Int J Mol Med* **2011**, *27*, 3–14. DOI: 10.3892/ijmm.2010.562	In vitro
Yang, L. C.; Lin, S. W.; Li, I. C.; Chen, Y. P.; Tzu; S. Y.; Chou, W.; Chen, C. C.; Lin, W. C.; Chen, Y. L.; Lin, W. H. *Lactobacillus* plantarum GKM3 and Lactobacillus paracasei GKS6 Supplementation Ameliorates Bone Loss in Ovariectomized Mice by Promoting Osteoblast Differentiation and Inhibiting Osteoclast Formation. *Nutrients* **2020**, *12*, 1914. DOI: 10.3390/nu12071914	Animal study/probiotics
Zheng, X. Q.; Huang, J.; Yuan, W. Q.; Wu, T.; Wang, H.; Liu, H.; Zhang, Y. D.; He, J. W.; Huang, C.; Song, C. L. Gut microbiota preserves bone mass through modulating the hyodeoxycholic acid-TGR5 axis. *Gut Microbes* **2025**, *17*, 2593088. DOI: 10.1080/19490976.2025.2593088	In vitro/animal study/ postbiotics
Zhou, Y.; Yang, Y.; Zhu, W.; Kourkoumelis, N.; Wang, Y.; Chen, Y.; Hong, L.; Wang, J.; Zhu, J.; Zhu, C.; Zhang, X. Microbial Influences on Calcium-Phosphorus Homeostasis and Metabolic Bone Diseases: A Bidirectional Mendelian Randomisation Study on the Gut-Bone Axis. *Cell Mol Med* **2025**, *29*, e70491. DOI: 10.1111/jcmm.70491	mendelian randomisation
**Review articles**	**Type of review**
Abu-Amer, Y. NF-κB signaling and bone resorption. *Osteoporos Int* 2013, 24, 2377-2386. DOI: 10.1007/s00198-013-2313-x	narrative
Baim, S.; Blank, R. Approaches to Fracture Risk Assessment and Prevention. *Curr Osteoporos Rep* 2021, *19*, 158-165. DOI: 10.1007/s11914-021-00659-x	narrative
Bolamperti, S.; Villa, I; Rubinacci, A. Bone remodelling: an operational process ensuring survival and bone mechanical competence]. *Bone Res* **2022**, *10*, 48. DOI: 10.1038/s41413-022-00219-8	narrative
Cheng, M.; Zhao, Y.; Cui, Y.; Zhong, C.; Zha, Y.; Li, S.; Cao, G.; Li, M.; Zhang, L.; Ning, K.; Han, J. Stage-specific roles of microbial dysbiosis and metabolic disorders in rheumatoid arthritis. *Ann Rheum Dis* 2022, 81,1669-1677. DOI: 10.1136/ard-2022-222871	narrative
Collins, S. L.; Stine, J. G.; Bisanz, J. E.; Okafor, C. D.; Patterson, A. D. Bile acids and the gut microbiota: metabolic interactions and impacts on disease. *Nat Rev Microbiol* **2023**, *21*, 236–247. DOI: 10.1038/s41579-022-00805-x	narrative
Ghorbani, Z.; Shoaibinobarian, N.; Noormohammadi, M.; Taylor, K.; Kazemi, A.; Bonyad, A.; Khoshdooz, S.; Löber, U.; Forslund-Startceva, S. K. Reinforcing gut	systematic

integrity: A systematic review and meta-analysis of clinical trials assessing probiotics, synbiotics, and prebiotics on intestinal permeability markers. *Pharmacol Res* **2025**, *216*, 107780. DOI: 10.1016/j.phrs.2025.107780	
Guan, Z.; Luo, L.; Liu, S.; Guan, Z.; Zhang, Q.; Li, X.; Tao, K. The Role of Depletion of Gut Microbiota in Osteoporosis and Osteoarthritis: A Narrative Review. *Front Endocrinol* **2022**, *13*, 847401. DOI: 10.3389/fendo.2022.847401	narrative
Gundberg, C. M.; Lian, J. B.; Booth, S. L. Vitamin K-dependent carboxylation of osteocalcin: friend or foe? *Adv nutr* **2012**, *3*, 149–157. DOI: 10.3945/an.112.001834	narrative
Harini, J.N.; Gayarthi, G.; Mahadevan, S.; Ilangovan, R. Effects of probiotic supplements on bone mineral density and bone turnover markers in postmenopausal women: A systematic review. *Clin Nutr ESPEN* **2025**, *69*, 503–515. DOI: 10.1016/j.clnesp.2025.07.1117	systematic
Hou, K.; Wu, Z.X.; Chen, X.Y.; Wang, J. Q.; Zhang, D.; Xiao, C.; Zhu, D.; Koya, J. B.; Wei, L.; Li, J.; Chen, Z. S. Microbiota in health and diseases. *Sig Transduct Target Ther* 2022, 7, 135. DOI: 10.1038/s41392-022-00974-4	narrative
Indrio, F.; Salatto, A. Gut Microbiota-Bone Axis. *Ann Nutr Metab* **2025**, *81(Suppl 1)*, 47-56. DOI: 10.1159/000541999	narrative
Keyvan, E.; Adesemoye, E.; Champomier-Vergès, M. C.; Chanséaume-Bussiere, E.; Mardon, J.; Nikolovska Nedelkoska, D.; Palamutoglu, R.; Russo, P.; Sarand, I.; Songre-Ouattara, L.; Trajkovska, B.; Karakaya, S.; Syrpas, M.; Chassard, C.; Pracer, S.; Vergères, G.; Heine, D.; Humblot, C. Vitamins formed by microorganisms in fermented foods: effects on human vitamin status-a systematic narrative review. *Front nutr* **2025**, *12*, 1653666. DOI: 10.3389/fnut.2025.1653666	systematic/narrative
Kim, C.H. Control of lymphocyte functions by gut microbiota-derived short-chain fatty acids. *Cell Mol Immunol* **2021**, *18*, 1161-1171. DOI: 10.1038/s41423-020-00625-0	narrative
Liu, S.; Li, G.; Xu, H.; Wang, Q.; Wei, Y.; Yang, Q.; Xiong, A.; Yu, F.; Weng, J.; Zeng, H. "Cross-talk" between gut microbiome dysbiosis and osteoarthritis progression: a systematic review. *Front Immunol* **2023**, *14*, 1150572. DOI: 10.3389/fimmu.2023.1150572	systematic
Locantore, P.; Del Gatto, V.; Gelli, S.; Paragliola, R. M.; Pontecorvi A. The interplay between immune system and microbiota in osteoporosis. *Mediators Inflamm* **2020**, *2020*, 3686749. DOI: 10.1155/2020/3686749	narrative
Ma, H.; Wang, K.; Jiang, C. Microbiota-derived bile acid metabolic enzymes and their impacts on host health. *Cell Insight* **2025**, *4*, 100265. DOI: 10.1016/j.cellin.2025.100265	narrative
Mandatori, D.; Pelusi, L.; Schiavone, V.; Pipino, C.; Di Pietro, N.; Pandolfi, A. The Dual Role of Vitamin K2 in "Bone-Vascular Crosstalk": Opposite Effects on Bone Loss and Vascular Calcification. *Nutrients* **2021**, *13*, 1222. DOI: 10.3390/nu13041222	narrative
Mukhopadhya, I.; Louis, P. Gut microbiota-derived short-chain fatty acids and their role in human health and disease. *Nat Rev Microbiol* 2025, *23*, 635–651. DOI: 10.1038/s41579-025-01183-w	narrative
Parada Venegas, D.; De la Fuente, M. K.; Landskron, G.; González, M. J.; Quera, R.; Dijkstra, G.; Harmsen, H. J. M.; Faber, K. N.; Hermoso, M. A. Short Chain Fatty Acids (SCFAs)-Mediated Gut Epithelial and Immune Regulation and Its Relevance for Inflammatory Bowel Diseases. *Front Immunol* **2019**, *10*, 277. DOI: 10.3389/fimmu.2019.00277	narrative
Park, E.; Ciofani, M. Th17 cell pathogenicity in autoimmune disease. *Exp Mol Med* **2025**, *57*, 1913–1927. DOI: 10.1038/s12276-025-01535-9	narrative
Priyadarshini, M.; Kotlo, K.U.; Dudeja, P.K.; Layden, B.T. Role of Short Chain Fatty Acid Receptors in Intestinal Physiology and Pathophysiology. *Compr Physiol* **2018**, *18*, 1091-1115. DOI: 10.1002/cphy.c170050	narrative

Qi, P.; Xie, R.; Liu, H.; Zhang, Z.; Cheng, Y.; Ma, J.; Wan, K.; Xie, X. Mechanisms of gut homeostasis regulating Th17/Treg cell balance in PMOP. *Front Immunol* **2024**, *15*, 1497311. DOI: 10.3389/fimmu.2024.1497311	narrative
Rashed, F.; Kamijyo, S.; Shimizu, Y.; Hirohashi, Y.; Khan, M.; Sugamori, Y.; Murali, R.; Aoki, K. The Effects of Receptor Activator of NF-κB Ligand-Binding Peptides on Bone Resorption and Bone Formation. *Front Cell Dev Biol* **2021**, *9*, 648084. DOI: 10.3389/fcell.2021.648084	narrative
Razzaque M. S. Interactions between FGF23 and vitamin D. *Endocr Connect* **2022**, *11*, e220239. DOI: 10.1530/EC-22-0239	narrative
Resch, H.; Zendeli, A.; Kocijan, R. Metabolic Bone Diseases-A Topic of Great Diversity. *J Clin Med* 2022, *11*, 6447. DOI: 10.3390/jcm11216447	narrative
Saadh, M. J.; Allela, O. Q. B.; Ballal, S.; Mahdi, M. S.; Chahar, M.; Verma, R.; Al-Hussein, R. K. A.; Adil, M.; Jawad, M. J.; Al-Nuaimi, A. M. A. The effects of microbiota-derived short-chain fatty acids on T lymphocytes: From autoimmune diseases to cancer. *Semin Oncol* **2025**, *52*, 152398. DOI: 10.1016/j.seminoncol.2025.152398	narrative
Shen, Y.; Fan, N.; Ma, S. X.; Cheng, X.; Yang, X.; Wang, G. Gut Microbiota Dysbiosis: Pathogenesis, Diseases, Prevention, and Therapy. *MedComm (2020)* **2025**, *6*, e70168. DOI: 10.1002/mco2.70168	narrative
Song, P.; Zhang, X.; Feng, W.; Xu, W.; Wu, C.; Xie, S.; Yu, S.; Fu, R. Biological synthesis of ursodeoxycholic acid. Front. Microbiol. **2023**, *14*, 1140662. DOI: 10.3389/fmicb.2023.1140662	narrative
Sun, J.; Zhang, Y. G. Vitamin D Receptor Influences Intestinal Barriers in Health and Disease. *Cells* **2022**, *11*, 1129. DOI: 10.3390/cells11071129	narrative
Tangestani, H.; Boroujeni, H. K.; Djafarian, K.; Emamat, H.; Shab-Bidar, S. Vitamin D and The Gut Microbiota: a Narrative Literature Review. *Clin Nutr Res* **2021**, *10*, 181–191. DOI: 10.7762/cnr.2021.10.3.181	narrative
Ulluwishewa, D.; Anderson, R. C.; McNabb, W. C.; Moughan, P. J.; Wells, J. M.; Roy, N. C. Regulation of tight junction permeability by intestinal bacteria and dietary components. *J Nutr* **2011**, *141*, 769–776. DOI: 10.3945/jn.110.135657	narrative
Wang, F., Wei, W.; Liu, P.J. Effects of probiotic supplementation on bone health in postmenopausal women: a systematic review and meta-analysis. *Front Endocrinol (Lausanne)* 2024, *15*, 1487998. DOI: 10.3389/fendo.2024.1487998	systematic/probiotics
Wang, J.; Zhu, N.; Su, X.; Gao, Y.; Yang, R. Gut-Microbiota-Derived Metabolites Maintain Gut and Systemic Immune Homeostasis. *Cells* **2023**, *12*, 793. DOI: 10.3390/cells12050793	narrative
Wang, Y.; Nishida, S.; Elalieh, H.Z.; Long, R.K.; Halloran, B.P.; Bikle D.D. Role of IGF-I signaling in regulating osteoclastogenesis. *J Bone Miner Res* **2006**, *21*, 1350-1358. DOI: 10.1359/jbmr.060610	narrative
Whisner, C. M.; Castillo, L. F. Prebiotics, Bone and Mineral Metabolism. *Calcif Tissue Int* **2018**, *102*, 443–479. DOI: 10.1007/s00223-017-0339-3	narrative
Wu, Y.; Yang, Y.; Wang, L.; Chen, Y.; Han, X.; Sun, L.; Chen, H.; Chen, Q. Effect of Bifidobacterium on osteoclasts: TNF-α/NF-κB inflammatory signal pathway-mediated mechanism. *Front Endocrinol (Lausanne)* **2023**, *14*, 1109296. DOI: 10.3389/fendo.2023.1109296	narrative
Xiang, T.; Deng, Z.; Yang, C.; Tan, J.; Dou, C.; Luo, F.; Chen, Y. Bile acid metabolism regulatory network orchestrates bone homeostasis. *Pharmacol Res* **2023**, *196*, 106943. DOI: 10.1016/j.phrs.2023.106943	narrative
Yadav, S.; Sapra, L.; Srivastava, R.K. Polysaccharides to postbiotics: Nurturing bone health via modulating "gut-immune axis". *Int J Biol Macromol* **2024**, *278*, 134655. DOI: 10.1016/j.ijbiomac.2024.134655	narrative
Yan, J.; Charles, J.F. Gut Microbiota and IGF-1. *Calcif Tissue Int* **2018**, *102*, 406-414. DOI: 10.1007/s00223-018-0395-3	narrative
Yao, D.; Huang, L.; Ke, J.; Zhang, M.; Xiao, Q.; Zhu, X. Bone Metabolism Regulation: Implications for the Treatment of Bone Diseases. *Biomed Pharmacother* **2020**, *129*, 110494. DOI: 10.1016/j.biopha.2020.110494	narrative

You, X.; Huang, D.; Zhang, Y.; Zboinski, E.; Hodys, C.; Tsang,K.; Charles, J.F. SCFA-GPR43 Axis Mediates Gut Microbiota Promotion of Juvenile Bone Growth. *Curr Develop Nutr* **2025**, *9*, 107182. DOI: 10.1177/1747493018778713	narrative
